# Unveiling the unseen: navigating heart involvement in amyloidosis with biomarkers beyond the horizons of imaging

**DOI:** 10.1093/ehjimp/qyag098

**Published:** 2026-05-28

**Authors:** Giuseppe Damiano Sanna, Paolo Milani, Valeria Anna Di Simone, Valentina Carini, Mario Nuvolone, Gavino Casu, Giovanni Palladini

**Affiliations:** Department of Molecular Medicine, University of Pavia, Viale Golgi, 19, Pavia 27100, Italy; Amyloidosis Research and Treatment Center, Fondazione IRCCS Policlinico San Matteo, Viale Golgi 19, Pavia 27100, Italy; Clinical and Interventional Cardiology, Sassari University Hospital, Via Enrico De Nicola, Sassari 07100, Italy; Department of Molecular Medicine, University of Pavia, Viale Golgi, 19, Pavia 27100, Italy; Amyloidosis Research and Treatment Center, Fondazione IRCCS Policlinico San Matteo, Viale Golgi 19, Pavia 27100, Italy; Amyloidosis Research and Treatment Center, Fondazione IRCCS Policlinico San Matteo, Viale Golgi 19, Pavia 27100, Italy; Division of Cardiology, Fondazione IRCCS Policlinico San Matteo, Viale Golgi 19, Pavia 27100, Italy; Amyloidosis Research and Treatment Center, Fondazione IRCCS Policlinico San Matteo, Viale Golgi 19, Pavia 27100, Italy; Division of Cardiology, Fondazione IRCCS Policlinico San Matteo, Viale Golgi 19, Pavia 27100, Italy; Department of Molecular Medicine, University of Pavia, Viale Golgi, 19, Pavia 27100, Italy; Amyloidosis Research and Treatment Center, Fondazione IRCCS Policlinico San Matteo, Viale Golgi 19, Pavia 27100, Italy; Clinical and Interventional Cardiology, Sassari University Hospital, Via Enrico De Nicola, Sassari 07100, Italy; Department of Molecular Medicine, University of Pavia, Viale Golgi, 19, Pavia 27100, Italy; Amyloidosis Research and Treatment Center, Fondazione IRCCS Policlinico San Matteo, Viale Golgi 19, Pavia 27100, Italy

**Keywords:** light-chain amyloidosis, transthyretin cardiac amyloidosis, biomarkers, natriuretic peptides, cardiac troponins, imaging

## Abstract

Amyloidosis refers to a heterogenous group of systemic diseases characterized by the presence of a misfolded protein that deposits as amyloid fibrils in the interstitium of organs and tissues. The vast majority of cases of cardiac involvement (cardiac amyloidosis—CA) are due to immunoglobulin light chain (AL) amyloidosis, or transthyretin (ATTR) amyloidosis (either in its wild-type form—ATTRwt, or variant—ATTRv). The diagnostic workup of these diseases reflects the differences in terms of aetiology. Although imaging techniques represent fundamental tools in the diagnosis and follow-up of patients CA, they present several limitations. Cardiac biomarkers, particularly natriuretic peptides and troponins, overcome most of these limitations. They can be more sensitive in the detection of early phases of the disease but, overall, they currently represent the most powerful tool to define disease stage, organ response and disease progression. There is mounting evidence regarding the use of specific laboratory biomarkers to monitor treatment response. The information provided by advanced imaging techniques should be regarded as complementary and not as substitute for that provided by laboratory biomarkers, as only these can often be able to unveil the unseen.

## Introduction

Systemic amyloidoses are caused by misfolded proteins that deposits as fibrils in organs and tissues.^1^ Potentially any organ can be involved with the heart being the key determinant of survival.^1^ The vast majority of cases of cardiac involvement is due to immunoglobulin light chain (AL) amyloidosis, or transthyretin (ATTR) amyloidosis (either wild-type—ATTRwt or variant—ATTRv). AL amyloidosis is caused by a plasma cell (PC) clone.^1^ Transthyretin (TTR) is primarily synthesized by the liver and circulates as a tetramer transporting thyroxin and retinol. TTR may dissociate into monomers and deposits as amyloid fibrils, either because of mutations that reduce the stability of its tetramers (ATTRv) or as an age-related phenomenon (ATTRwt). Despite some similarities, AL and ATTR amyloidosis with cardiac involvement (AL-CA and ATTR-CA) are different entities and should not be regarded simply as distinct phenotypes of the same disease. The diagnostic workup reflects these differences, and it first requires (in patients with suspicious clinical and/or instrumental findings) the search for a monoclonal protein (MP) by immunofixation of serum and urine and measurement of circulating free light chains (FLCs).^1,2^ This step is crucial to exclude AL amyloidosis, which can be fatal within a few weeks/months in case of diagnostic delays.^3^ The presence of a MP always requires a histological confirmation (affected organ or surrogate site) to achieve a definite diagnosis of AL amyloidosis or to exclude it.^1^ In patients without MP (and thus without AL-CA), the final diagnosis (of ATTR-CA—either ATTRwt or ATTRv—or rarer hereditary forms) can be obtained, in most cases, through a non-invasive algorithm based on scintigraphy with bone tracers and genetic testing.^4^

Considered rare diseases until just over a decade ago, amyloidoses now represent a hot topic in the field of heart failure (HF), cardiomyopathies and cardiovascular imaging. This change is probably due to the availability of efficacious disease-modifying treatments and the wide diagnostic horizons provided by non-invasive cardiovascular imaging.^4,5^

However, the inappropriate use of imaging techniques without integration with clinical and laboratory data (including biomarkers) often contributes to generate a diagnostic mist which in turns may negatively affect the diagnostic workup and overall patients’ management and outcomes.^3^ In this review, we analyse the role of biomarkers in the diagnosis and management of patients with cardiac amyloidosis (CA), since they often represent the ‘*Maid of the Mist*’ in a journey somewhat resembling the tour of Niagara Falls.

## Discussion

### Pathophysiological mechanisms underlying the increase of biomarkers

In CA, the mechanisms of organ damage and dysfunction are likely multifactorial.^6^ Mechanical displacement of normal parenchymal tissue by amyloid deposits is insufficient to fully explain organ dysfunction.^6^ Experimental and clinical evidence indicate a discrepancy between amyloid burden and the grade of cardiac impairment and overall aggressive disease trajectory in patients with AL amyloidosis compared with those with ATTR-CA.^6^ In AL amyloidosis, prefibrillar cardiotropic light chains (LCs) induce a marked increase of intracellular reactive oxygen species, which leads to increased oxidative stress and apoptosis.^6^ Oxidant stress results in direct impairment of cardiomyocyte contractility and relaxation with abnormal intracellular calcium handling.^6^ The activation of p38 MAPK (mitogen-activated protein kinase) is one of the molecular mechanisms responsible for cardiotoxicity by increasing oxidative stress and apoptosis.^7^ This pathway also mediates B-type natriuretic peptide (BNP) transcription, supporting the association between cardiotoxic LC effects and elevated BNP levels.^7^ Exposure of cardiac fibroblasts to cardiotropic immunoglobulin LCs translates into proteome remodelling affecting proteins involved in cytoskeletal organization, protein synthesis and quality control, mitochondrial activity and metabolism, signal transduction and molecular trafficking.^6^ Regarding ATTR-CA *in vitro* experiments suggest that TTR monomers and oligomeric intermediates, but not large aggregates or amyloid fibrils, induce cytotoxicity through interactions with membrane proteins and cholesterol. Apoptotic mechanisms are activated through cleavage of caspase 3/7 and superoxide formation.^6^ Inflammation represents another piece of the pathophysiological puzzle of CA.^8^ Almost 50% of patients with CA showed intramyocardial inflammation that contributes to increased mortality. In addition, one-third of patients with ATTR-CA exhibit signs of myocardial inflammation that may be associated with increased risk of death and HF hospitalizations.^9^ Therefore, intramyocardial inflammation is not a hallmark specific of AL amyloidosis. There are probably significant differences in the underlying mechanisms of intramyocardial inflammation between AL-CA and ATTR-CA, and they are reflected by cardiovascular imaging. For example, native T1 is higher in AL-CA compared to ATTR-CA as a possible result of more hydration of the amyloid, more collagen associated with amyloid, differential effects on the intracellular signal, and oedema due to LCs toxicity.^5^ In this complex scenario of myocardial inflammation, macrophages seem to play a pivotal role.^10^ Coronary microvascular dysfunction also contributes to myocardial injury. Amyloid deposits can be found in the perivascular regions and in the media of intramyocardial coronary vessels.^11^

**Figure 1:**
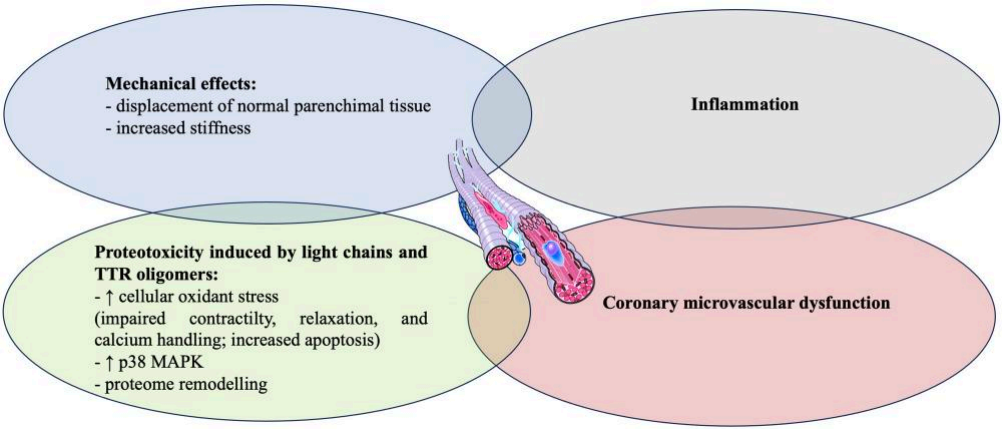
Pathophysiological mechanisms underlying the increase of cardiac biomarkers in systemic amyloidoses.

All the aforementioned mechanisms contribute to the significant increase of cardiac biomarkers (i.e. natriuretic peptides—NPs and cardiac troponins—cTn) observed in CA, as summarized in *Figure 1*.

### Role of biomarkers in the diagnosis of cardiac involvement

Although at present time, the role of biomarkers is somewhat hidden in the diagnostic algorithm for CA, both NPs and cTn have a pivotal role in the diagnostic workup of patients.^1^ If on the one hand, values still within normal range can be found in patients with ATTR-CA but without HF symptoms despite an overt instrumental phenotype (and this is due to the increased awareness of the disease thanks also to cardiovascular imaging with the possibility to intercept the disease at earlier stages), biomarkers (particularly serum N-terminal pro-B-type natriuretic peptide—NT-proBNP) still represent the most sensitive and powerful tool to identify myocardial involvement in AL-CA.^12^ Moreover, a biomarker-based screening using NT-proBNP in patients with monoclonal gammopathy of undetermined significance (MGUS) can raise the suspicion and identify initial pre-symptomatic organ involvement.^13^ However, despite highly sensitive in detecting pre-symptomatic AL amyloidosis in this setting, biomarkers lack specificity, and further studies on the cost/benefit ratio of this approach are needed before recommending it as standard practice. NPs and cTn can also act as gatekeepers in the follow-up of patients with confirmed AL amyloidosis without cardiac involvement at baseline diagnosis, to promptly detect its appearance.

Despite the undoubted power of NPs and cTn in the diagnostic workup of CA, some clinical scenarios remain challenging, even when cardiac biomarkers are used in combination with non-invasive imaging tests; these include, for example, patients with low estimated glomerular filtration rate—eGFR (i.e. <30 mL/min/1.73 m^2^) and borderline/mildly increased cardiac wall thickness. On the one hand, these patients usually exhibit higher values of both NPs and cTn; on the other hand, gadolinium contrast agents cannot be administered due to the potential risk of developing nephrogenic systemic fibrosis.^14,15^ This limits the diagnostic capacity of cardiovascular magnetic resonance (CMR); in fact, an increase of native T1 can be observed in several conditions (e.g. hypertensive heart disease, hypertrophic cardiomyopathy, and CA without a clear distinction between AL-CA and ATTR-CA), and this does not allow for a differential diagnosis when the study of late gadolinium enhancement is not performed. Modest chronic increases in cardiac biomarkers can be observed in patients with chronic ischaemic heart disease, rhythm disorders, and other cardiac conditions. When facing these clinical scenarios, biomarkers within normal reference range despite instrumental red flags of the disease are probably able to ruling-out AL-CA in most of the cases (but not so effectively ATTR-CA);^16^ however, in patients with increased cardiac biomarkers, a definite diagnosis of CA remains a challenge with the clinician being between Scylla and Charybdis, balancing the choice between deferring the diagnosis of a potentially-life threatening disease and the risks of an organ biopsy in often frail and elderly subjects. The integration of the information provided by cardiac biomarkers with CMR data may help orient the bow of the boat towards the correct definition of cardiac phenotype. According to the diagnostic algorithm proposed in the position statement of the European Society of Cardiology (ESC) Working Group on Myocardial and Pericardial diseases CMR, apart from its role in raising the suspicion of CA, can be used afterwards when the results of haematologic tests and of bone scintigraphy are both negative or when the latter is negative in the presence of a MP with clinical and instrumental red flags suggestive of CA. In these cases, a CMR is recommended, followed by biopsy based on its results.^17^ However, this diagnostic approach, useful in some cases, also to prevent unnecessary and harmful organ biopsies (and potentially able to catch the potential missed diagnoses of ATTRv-CA due to certain variants by bone scintigraphy without proper genetic testing), is much less emphasized in the diagnostic algorithm proposed in the USA.^1^ Moreover, it should be kept in mind that at present time, CMR should neither replace nor defer histological confirmation.^1,3^

As previously stated, when biomarkers fall within normal reference range despite instrumental red flags of the disease ruling-out ATTR-CA can be a challenge, as shown by a retrospective study in patients undergoing Technetium Tc 99m pyrophosphate scintigraphy in which only very low levels [i.e. < 6 ng/L] of hs-cTnT were able to effectively rule-out the diagnosis with higher thresholds increasing the rate of false negatives [NT-proBNP and combined biomarkers strategies showed similar trends].^16^ According to another study involving three tertiary centres, the diagnosis of CA can be reliably excluded when NT-proBNP is <180 ng/L and hs-TnT < 14 ng/L.^18^ The reason for these discrepancies between studies is probably due to the prevalence of the disease in the studied cohorts and the pretest probability.^16,18^

Cardiac biomarkers (namely hs-cTnI/T) may represent the core of a screening strategy to reveal early cardiac involvement in carriers of pathogenic TTR variants. According to a recently proposed screening algorithm, hs-cTn could be measured beginning 10 years before the predicted age of disease-onset (PADO); if hs-cTn remains stable or increases <50% in 1 year, another measurement after 1 year may suffice. A hs-cTn value that increases ≥50% in 1 year in the absence of possible explanatory causes suggests myocardial damage related to amyloid deposition. This may warrant searching for extracellular volume (ECV) expansion or regional bone tracer uptake via CMR or single-photon emission computed tomography with bone tracer, respectively.^19^

According to recent data, CMR-derived ECV seems to be able to quantify myocardial amyloid load in ATTR amyloidosis: ECV <30% reliably excludes cardiac involvement (99% sensitivity), while an ECV ≥40% confirms overt ATTR-CA (99% specificity).^20^ However, these data derive from a single-centre retrospective study, and this limits generalizability.

### Role of biomarkers in the staging of cardiac involvement

Cardiac biomarkers represent the core of current staging systems for both AL-CA and ATTR-CA (*Table 1*).

**Table 1:** Role of cardiac biomarkers in the diagnosis and management of patients with AL-CA and ATTR-CA

	Diagnosis (early/preclinical)	Diagnosis	Staging	Disease progression	Treatment and organ response
**AL-CA**	**Natriuretic peptides** NT-proBNP > 332 ng/L in patients with intermediate/high-risk MGUS **Cardiac troponins** Undefined	**Natriuretic peptides** NT-proBNP > 332 ng/L **Cardiac troponins** hs-TnT ≥ 35 ng/L hs-TnI ≥ 100 ng/L	**Mayo (European rev.)** **Stage I**—both biomarkers below the cutoffs; **Stage II**—one biomarker is elevated; **Stage IIIa**—both biomarkers are elevated but NT-proBNP is < 8.500 ng/L; **Stage IIIb**—both biomarkers are elevated with NT-proBNP > 8.500 ng/L	**Organ disease progression** NT-proBNP increase > 30% and > 300 ng/L OR cTn increase ≥ 33%, OR ≥ 10% decrease in LVEF	**Hematologic response** Complete response—CR (defined by negative sIFE and uIFE + either a FLC ratio within the reference range or the uninvolved FLC concentration greater than involved FLC concentration with or without an abnormal FLC ratio); Very good partial response—VGPR (dFLC [difference between the involved and uninvolved light chains] < 40 mg/L); Partial response—PR (dFLC decrease > 50% compared to baseline); No response—NR (all other patients) **Organ response** **Natriuretic peptides** NT-proBNP decrease > 30% and > 300 ng/L in patients with baseline levels ≥ 650 ng/L OR ≥ 2 class decrease in the NYHA class in those with baseline NYHA III or IV) **Grading cardiac response** CarCR (nadir NT-proBNP ≤ 350 ng/L or BNP ≤ 80 ng/L); CarVGPR (> 60% reduction in NT-proBNP/BNP from baseline level not meeting CarCR); CarPR (31–60% reduction in NT-proBNP not meeting CarCR); CarNR (≤30% reduction in NT-proBNP not meeting CarCR) **Cardiac troponins** Undefined
**ATTR-CA** **(ATTRwt and ATTRv)**	**Natriuretic peptides** ^a^ NT-proBNP < 180 ng/L (to rule-out) **Cardiac troponins** ^a^ hs-TnT < 14 ng/L (to rule-out) hs-TnT ≥ 86 ng/L (to rule-in) In ATTRv:** Annual measurement of hs-Tn beginning 10 years before PADO. An hs-cTn value that increases ≥50% in 1 year in the absence of possible explanatory causes (e.g. worsening renal function) suggests myocardial damage related to amyloid deposition.	**Natriuretic peptides** ^a^ NT-proBNP < 180 ng/L (to rule-out) **Cardiac troponins** ^a^ hs-TnT < 6 ng/L in patients undergoing ^99m^Tc PYP imaging (to rule-out) hs-TnT < 14 ng/L (to rule-out) hs-TnT ≥ 86 ng/L (to rule-in)	**NAC staging** **Stage I**—NT-proBNP ≤ 3000 ng/L and eGFR ≥ 45 mL/min; **Stage II**—NT-proBNP ≥ 3000 ng/L or eGFR ≤ 45 mL/min; **Stage III**—those with both NT-proBNP ≥ 3000 ng/L and eGFR ≤ 45 mL/min **Mayo staging** **Stage I—**Troponin T < 50 ng/L and NT-proBNP < 3000 ng/L **Stage II—**Troponin T > 50 ng/L or NT-proBNP > 3000 ng/L **Stage III**—Troponin T > 50 ng/L and NT-proBNP > 3000 ng/L **Columbia** Further refines NAC and Mayo Clinic staging systems by adding the diuretic dose and the NYHA functional class **Others** hs-cTnI > 80 ng/L is able to predict all-cause mortality both in isolation and together with natriuretic peptides (i.e. > 3000 ng/L for NT-proBNP and > 250 ng/L for BNP).	**Natriuretic peptides** An increase in NT-proBNP >700 ng/L and >30%+ any initiation or up-titration of loop diuretics (ODI) **Cardiac troponins** Undefined **Expert consensus ESC 2021** Set of 11 measurable features across three separate domains: Clinical and functional endpoints (HF hospitalizations, OR increase in the NYHA class, OR decline in quality of life assessed through KCCQ [5–10 pts] or EQ-5D [10%] OR 30–40 m decline in 6MWT every 6 months); Laboratory biomarkers (30% increase in NT-proBNP [300 ng/L cutoff], OR 30% increase in troponin, OR advance in NAC staging scale); Imaging and ECG (increased LV wall thickness [2 mm], OR increase in diastolic dysfunction grade, OR change in systolic measurement [≥5% decrease in LVEF; ≥ 5 mL decrease in stroke volume; ≥ 1% increase in GLS], OR new-onset conduction disturbance). One marker from each of the three domains provides the minimum requirements for assessing disease progression. **Expert update 2026** The updated proposed criteria include 6 parameters, with corresponding thresholds and monitoring frequency (12 mo) recommendations. Category: clinical Heart failure hospitalization (≥ 1 event) ODI Category: biomarker NT-proBNP (increase > 700 ng/L and relative increase > 30%) eGFR (relative decrease > 20%) Category: functional 6MWT (decrease > 35 meters) KCCQ (> 5-point decrease) The NYHA (increase in class)	**Natriuretic peptides** Undefined **Cardiac troponins** Undefined

AL-CA, light chain amyloidosis with cardiac involvement; ATTR-CA, transthyretin amyloidosis with cardiac involvement; ATTRwt, wild-type transthyretin amyloidosis; ATTRv, hereditary transthyretin amyloidosis; NT-proBNP, N-terminal pro-B-type natriuretic peptide; MGUS, monoclonal gammopathy of undetermined significance; low-risk MGUS—according to Mayo risk stratification (i.e. monoclonal protein—MC ≤ 1.5 g/dL; Immunoglobulin G [IgG] type, normal free light chain ratio—FLCr); intermediate/high-risk MGUS—according to Mayo risk stratification (all the others); hs-TnT, high sensitivity troponin T; hs-TnI, high sensitivity troponin I; PADO, predicted age of disease-onset; ^99m^Tc PYP imaging, Technetium Tc 99m pyrophosphate scintigraphy; eGFR, estimated glomerular filtration rate; NYHA, New York Heart Association; BNP, brain natriuretic peptide; ODI, outpatient diuretic intensification; KCCQ, Kansas City Cardiomyopathy Questionnaire; 6MWT, 6-minute walk test; KCCQ—sIFE, serum protein electrophoresis + immunofixation; uIFE, urine protein electrophoresis + immunofixation; FLC, serum kappa and lambda free light chains.

^a^Not yet universally accepted/proposed.

#### AL amyloidosis

The staging system reported by the Mayo Clinic group and subsequently modified by European groups, based on the combination of NT-proBNP and cardiac TnT or TnI at presentation, has become the standard for staging patients with AL-CA (*Table 1*).^21,22^ In 2012, the Mayo Clinic group revised the staging system incorporating the difference between involved and uninvolved FLC (dFLC).^23^ Subsequent studies suggested a better performance of the European modification of the Mayo 2004 staging system over the Mayo 2012 model.^24^

More recently, a new international staging system (AL-International Staging system [AL-ISS]) has been defined and validated. This system incorporates echocardiographic global longitudinal strain (GLS) into the biomarker-based European modification of the Mayo 2004 model, defining an ultra-poor risk group (Stage IIIc, *Table 1*).^25^ Although GLS performs well in this setting, there are potential limitation. GLS is a load-dependent measurement with significant differences between vendors which can make standardization difficult to achieve in clinical practice. Finally, intra- and inter-observer variability may be high.

#### ATTR amyloidosis

For patients with ATTR-CA (both ATTRwt and ATTRv amyloidosis), the most widely used staging system in clinical practice and randomized trials is the one proposed by the National Amyloidosis Centre (NAC), based on NT-proBNP and eGFR (*Table 1*).^26^

In 2016 researchers from the Mayo Clinic proposed another cardiac biomarker staging system that used thresholds of troponin T and NT-proBNP (*Table 1*).^27^

According to a recent observational multicentre study on patients with ATTRwt-CA, a hs-cTnI threshold of 80 ng/L (using different assays) was able to predict all-cause mortality at 18 months. The prognostic value was conformed when using the same threshold in a 2-variable staging system including NPs (i.e. > 3000 ng/L for NT-proBNP and > 250 ng/L for BNP) (*Table 1*).^28^

Finally, similarly to what has been observed in AL-CA, echocardiographic GLS (baseline cutoff value—12.8%) resulted a good prognosticator both in isolation and over baseline NAC stage in ATTRwt-CA (*Table 1*).^29^

### Role of biomarkers in the evaluation of disease progression and treatment response

#### AL amyloidosis

In AL-CA organ disease progression can be defined by NT-proBNP increase >30% and >300 ng/L OR cTn increase ≥33%, OR as a ≥10% decrease in left ventricular ejection fraction (LVEF).^30^

Response in AL amyloidosis includes assessment of the direct impact of chemotherapy on the clone and the indirect effect on organ function based on baseline organ damage as well as depth of haematologic response achieved. Although the deeper the haematologic response, the greater is the likelihood of organ response, there may be discrepancies between these two. Therefore, in AL-CA both the cardiologist and the haematologist must be aware of both the respective response criteria, in order to properly tailor and taper their therapies in the single patient. Definition of haematologic response in AL amyloidosis is reported in *Table 1*.^30^ Although organ response is the real target of treatment but none of available chemotherapy agents can influence this directly, achieving a haematologic complete response (CR) currently represents the goal of treatment in AL amyloidosis. The role of minimal residual disease (MRD) as a potential end-point for clinical trials such as in multiple myeloma is still under investigation.

The definition of cardiac response is reported in *Table 1*. Cardiac response tends to improve over time, and patients with cardiac complete response have survival rates similar to those of a matched general population.^31^ Some recent studies investigated the ability of CMR to measure changes in response to chemotherapy. A study comprising 221 patients with AL-CA suggested that a change in native T1 (i.e. ≥ 50 msec) may be used to track treatment response.^32^ More interestingly, in another study involving 176 patients with AL-CA, a ≥5% decrease in ECV (considered as a surrogate of amyloid deposits), was frequently observed only in patients who achieved CR/very good partial response -VGPR with chemotherapy (38% after 2 years). Moreover, changes in ECV predicted survival at 6 months and 1-year post-chemotherapy after adjusting for known predictors (i.e. haematological response, change in NT-proBNP and longitudinal strain); however, these changes were relatively uncommon and late.^33^ The rationale for using ECV as a surrogate marker of amyloid burden and to monitor treatment response is intriguing. However, it should be kept in mind that, apart from interstitial protein deposition and oedema, changes in vascular volume can affect ECV as well as changes in size of the intracellular volume (ECV is a volume fraction).^34^ Technical factors also represent another potential source of bias (e.g. the timing of phlebotomy and posture of the patients to determine haematocrit, the choice of the region of interest, etc.). In a recent prospective study including 81 patients with AL-CA using ^18^F-florbetapir positron emission tomography—PET/CT and CMR, molecular amyloid burden was estimated as ^18^F-florbetapir percentage injected dose (%ID) at baseline, and at 6 and 12 months after plasma-cell directed chemotherapy. The %ID decreased significantly among biomarkers responders, whereas there was no change in non-responders. Surprisingly, ECV did not change neither in biomarkers responders nor in non-responders. The Authors justified this finding stating that %ID and ECV probably reflect different pathophysiological aspects of myocardial involvement in AL-CA.^35^ All the aforementioned imaging-based studies emphasize the role of cardiac biomarkers (mainly NT-proBNP changes) as the currently available simplest method to define organ response in AL-CA, as well as a sort of ‘gold-standard’ method to compare with. Imaging is probably able to refine the information provided by cardiac biomarkers, but it does not seem to be able to replace their simplicity, diagnostic accuracy and cost-effectiveness so far.

#### ATTR amyloidosis

Regarding ATTR-CA disease progression, a few years ago an expert consensus of the ESC proposed the use of a set of 11 measurable features across three separate domains: I. Clinical and functional endpoints; II. Laboratory biomarkers; III. Imaging and ECG. Experts recommended that one marker from each of the three domains provides the minimum requirements for assessing disease progression (*Table 1*).^36^ However, this method (based on the consensus of experts) had some limitations. For example, it did not take into account the role of supporting HF medications, particularly outpatient diuretic intensification (ODI) which may potentially influence the variables included in all the three domains.^36^ Moreover, although the echocardiographic imaging thresholds for LVEF (≥5% decrease), stroke volume (≥5 mL decrease), and GLS (≥1% increase) were extracted from clinical trials, these parameters are highly load dependent and the threshold of interobserver variability can easily exceed the aforementioned cutoffs. Moreover, these small changes may not be above minimal clinically important differences.

The increasing availability of the so-called disease-modifying therapies for ATTR-CA has spurred an increased need to refine the criteria for disease progression. In this context, strong evidence supporting the prognostic role of biomarkers derives from a large multicentre retrospective study including 2275 patients with ATTR-CA (321[14%] treated with the TTR stabilizer tafamidis). At 12 months, an increase in NT-proBNP >700 ng/L and >30%, as well as any initiation or up-titration of loop diuretics (ODI), was independently predictive of all-cause mortality. The combination of these two parameters allowed for effective risk stratification.^37^

More data regarding disease progression in patients treated with disease-modifying drugs derived from an Italian multicentre longitudinal study involving 683 patients with ATTRwt-CA and the NYHA class I—II treated with tafamidis. Two predictive models were developed: one based on clinical progression to the NYHA class III and NAC stage worsening and a second model incorporating ODI, eGFR decline, and NT-proBNP elevation. Both models demonstrated comparable accuracy in predicting adverse outcomes.^38^ In light of the aforementioned evidence and developments, international experts recently proposed an update of the 2021 criteria (*Table 1*).^36^ It is worth noting the fact that, in contrast to the previously published criteria, imaging parameters were excluded, and six other parameters are now considered (clinical [HF-related hospitalizations, ODI], biomarkers [NT-proBNP, eGFR], functional [6-minute walk distance—6MWD, Kansas City Cardiomyopathy Questionnaire]).^36^ When considering ATTR-CA, we should acknowledge the fact that, despite the advent of disease-modifying drugs, the proof of their efficacy provided by clinical trials and the increasing availability of real-world data deriving from large registries, treatment response criteria are not defined. Although disease progression criteria should be regarded as the other side of the coin providing indirect information in terms of response to treatments, we still face with the absence of well-established parameters and scores to measure and define the response to disease-modifying therapies. In a recent study from the NAC of 189 patients with ATTR-CA treated with patisiran ECV progression was observed in untreated patients, while no significant difference in mean ECV was found in those treated with patisiran. Moreover, ECV progression was independently associated with mortality after adjusting for known predictors (i.e. increase in NT-proBNP > 700 ng/L and 30% AND ODI).^39^ Apart from some limitations (single-centre, possible selection bias—patients with severe renal impairment, cardiac implantable electronic devices, or difficulties to lie flat were excluded, treatment with patisiran expected to be replaced by vutrisiran, plus some potential technical issues regarding ECV measurement), this study might open an intriguing perspective, similarly to AL-CA.^39^ An attractive mechanistic hypothesis suggests that, in patients with ATTR amyloidosis, low serum TTR (sTTR) reflects enhanced tissue deposition and more intense end-organ involvement. Lower sTTR has been associated with increased levels of cardiac biomarkers (i.e. cTn-I), higher NAC stage, and higher risk of death from any cause.^40^ Circulating TTR levels change in response to treatment, with TTR-stabilizers increasing levels, and TTR gene-silencers reducing levels. Since the mechanism of action seems to be somewhat opposite, in order to avoid confusion and potential misleading explanations of complex biological actions, it should be kept in mind that TTR stabilizers work by stabilization of the circulating tetramer, impeding its dissociation into monomers that can generate toxic, oligomeric TTR amyloid precursors. Greater TTR stabilization manifests as reduced clearance and consequently increased sTTR levels. Recent data suggest that acoramidis, a novel, high-affinity stabilizer that achieves ≥90% TTR stabilization *in vitro*, is able to determine a sharp and significant early rise in sTTR levels within 28 days and sustained over time. The ΔTTR is independently associated with improved survival.^41^ These data suggest that sTTR levels might be used to track treatment response over time. Recently, in a cohort of 169 patients initiating tafamidis at 3 Italian centres, those with baseline sTTR < 18 mg/dL had significantly increased all-cause mortality, while blunted increase (i.e. ΔTTR < 7.5 mg/dL) predicted HF hospitalization.^42^ In another cohort of 185 tafamidis-treated patients from the Columbia University, an increase in sTTR levels was associated with an improved survival.^43^ It remains unclear whether a specific Δ in TTR levels can be deemed clinically meaningful and help differentiate responders from non-responders. In the use of TTR as a biomarker to monitor treatment response, we should face with several challenges; these include undefined thresholds and cutoffs (that’s why the use of Δ is probably more useful), and interference from nutritional status—often impaired in patients with amyloidosis ad HF—and inflammation being a negative acute phase protein.^44^ The possibility of an integration of serum TTR levels and imaging features should be explored in future studies. In a recent study investigating left ventriculo-arterial coupling (VAC) in a contemporary cohort of patients with ATTRwt-CA treated with the stabilizer tafamidis, serum TTR levels did not show any correlation with the echocardiographic-derived measurement of VAC.^45^ With a progressively more extensive use of disease-modifying drugs in ATTR-CA, there is growing evidence suggesting that a significant proportion of treated patients experience clinically meaningful improvement in terms of NP reduction and 6MWD increase; this might suggest that the treatment paradigm may evolve from slowing disease progression to achieving improvement.^46^ In the context of ATTRwt-CA, characterized on the one hand by an increase of new diagnoses accompanied by a complex decision-making when determining whether or not to treat patients who are often 80 years old and older, and on the other hand by the need to maintain economic sustainability, the finding of a true cost-benefit ratio is fundamental to guarantee treatment equity with an optimal management of healthcare system resources. The identification of patients at high risk of early death may allow clinicians to better and precisely identify those who may not benefit from slow-acting disease-modifying treatments, needing instead supporting therapies. A simple stratification model based on the combination of NT-proBNP and troponin levels, age, and overall performance status assessed through the Eastern Cooperative Oncology Group—Performance status (ECOG-PS) is able to identify patients with ATTRwt-CA at risk of early death (i.e. <18 months), and thus less-likely to benefit from slow-acting treatments.^47^ These data emphasize again the value of simple laboratory cardiac biomarkers over complex and more expensive imaging techniques.

### Role of biomarkers to guide HF supporting treatments

Amyloidosis with cardiac involvement is characterized by a labile equilibrium between preload and afterload.^1,45^ However, a precise evaluation of volume status in the single patient is difficult to obtain. Although in the GUIDE-IT study, a strategy of NT-proBNP-guided therapy was not more effective than a usual care in improving outcomes, and similar results were obtained in the PRIMA II trial in patients admitted due to acute decompensated HF (with a target of >30% NT-proBNP reduction from admission to discharge), NPs are still widely used in clinical practice to guide patients’ decongestion and diuretic prescription.^48,49^ However, this management strategy may have several shortcomings, especially in AL-CA. The increased levels of NPs in these patients do not simply represent a marker of congestion. Therefore, although serial evaluations of NPs are indicated to monitor organ response and disease progression, the interpretation of their levels should include several parameters: stability of patient’s symptoms and the NYHA class, body weight, overall fluid balance and restriction, physical signs of volume overload, chemotherapy regimens, heart rate and imaging findings (mainly echocardiographic—e.g. stroke volume, diastolic function—E/e′ ratio, pulmonary pressures—PASP, inferior vena cava size, pulmonary B-lines). Volume management in patients with CA is often empirical, without specific recommendations.^50^ The Venous Excess UltraSound (VExUS) protocol may help in diuretic therapy management in combination with laboratory biomarkers; however, this should be addressed in future studies.

### Emerging biomarkers

Apart from the established role of NPs and cardiac troponins, other biomarkers (e.g. of the activation of neurohormonal pathways) show potential usefulness both in the diagnosis and management of patients with CA.

During the diagnostic workup, hepatocyte growth factor (HGF) may be useful to distinguish AL-CA from other conditions with similar clinical and instrumental features. In fact, HGF levels are markedly elevated in patients with AL-Ca compared with those with ATTR-CA or left ventricular hypertrophy.^51^

Growth differentiation factor-15 (GDF-15) is a member of the transforming growth factor β family. Cardiac myocytes produce and secrete GDF-15 in response to oxidative stress, stimulation with angiotensin II or proinflammatory cytokines, ischaemia, and mechanical stretch. In patients with AL amyloidosis, a high GDF-15 level is associated with a higher risk of early death and poor overall survival independently of NT-proBNP and hsTnT or hsTnI levels.^52^

Endothelin-1 (ET-1) is a vasoconstrictive peptide that is primarily produced by endothelial cells and cardiomyocytes. It seems to be involved in the pathophysiology of HF, by causing cardiac hypertrophy and having profibrotic and proinflammatory effects. In a retrospective study involving 141 patients with newly diagnosed AL-CA, higher big ET-1 (the precursor of endothelial-vasoconstrictive ET-1) levels were a strong and independent predictor of mortality, slightly refining the modified Mayo staging system.^53^

The sST2 (soluble suppression of tumorigenesis-2) is a protein biomarker released in response to vascular congestion, inflammatory, and pro-fibrotic stimuli. This biomarker seems to have an independent prognostic role in AL amyloidosis but not in ATTR-CA.^54,55^

We have previously discussed about the potential role of TTR as a potential biomarker to monitor treatment response in ATTR-CA. TTR aggregate detector (TAD1) targets TTR segments exposed only in pathogenic conformations to detect ATTR fibrils and aggregates in patients’ tissues and blood.^56^ These preliminary experiences open the way for future applications not only for early ATTR detection but also for monitoring treatment response.^56^

The role of these biomarkers in the clinical arena is still incompletely defined, and further data and evidence regarding also their costs and availability are required before including them in routine clinical practice.

### Biomarkers beyond the horizons of cardiovascular imaging

The optimal integration of biomarkers and imaging remains to be defined, although some studies have already shown a potential success when combining the two (*Table 2*).^57^

**Table 2:** Integrative role of imaging in the diagnosis and management of patients with AL- and ATTR-CA

	Integrative role of imaging in the diagnosis	Integrative role of imaging in current staging systems	Integrative role of imaging in the assessment of disease progression and treatment response
**AL-CA**	**Echocardiography** No criteria/cutoffs (wall thickness, GLS) to define early phenotypic involvement in isolation	**Echocardiography** **AL- International Staging system [AL-ISS]** This system incorporates echocardiographic GLS into the biomarker-based European modification of the Mayo 2004 model, defining an ultra -poor risk group of patients (Stage IIIc). According to this system **Stage IIIb** is defined as GLS < −9%, NT-proBNP ≥ 8500 ng/L, hs-TnT ≥ 50 ng/L, while **IIIc** as GLS ≥ −9%, NT-proBNP ≥ 8500 ng/L, hs-TnT ≥ 50 ng/L	**Echocardiography** ^a^ (1) **Stringent cardiac response** (NT-proBNP decrease > 30% and > 300 ng/L AND GLS improvement >/=2%); (2) **Cardiac response** (NT-proBNP decrease > 30% and > 300 ng/L OR GLS improvement >/=2%); (3) **Stable cardiac disease** (NT-proBNP decrease < 30% or 300 ng/L OR GLS improvement <2%); (4) **Cardiac progression** (any worsening of NT-proBNP OR any worsening of GLS)
	**Cardiac magnetic resonance** No criteria/cutoffs (e.g. native T1, ECV) to define early phenotypic involvement in isolation	**Cardiac magnetic resonance** Undefined	**Cardiac magnetic resonance** ^a^ Change in T1 (i.e. Δ ≥50 msec) Change in ECV (≥5% decrease)
	**Nuclear imaging** ^a^ Promising results with [18F]-Florbetaben, Florbetapir PET/CT, and 124I-evuzamitide to differentiate between AL-CA, ATTR-CA and other mimicking conditions. *Echocardiography and cardiac magnetic resonance are fundamental to raise the suspicion but the diagnosis always requires histological confirmation.*		**Nuclear imaging** ^a^ ^18^F-florbetapir percentage injected dose (%ID) decrease in chemotherapy responders.
**ATTR-CA** **(ATTRwt and ATTRv)**	**Echocardiography** No criteria/cutoffs (wall thickness, GLS) to define early phenotypic involvement in isolation	**Echocardiography** ^a^ Baseline GLS (cutoff value −12.8%) able to predict all-cause mortality independently and over NAC biomarker staging	**Echocardiography** Undefined
	**Cardiac magnetic resonance** ECV < 30% to rule out (e.g. in TTR mutation carriers) ECV ³ 40% to rule in *Echo and CMR are fundamental to raise the suspicion of the disease.*	**Cardiac magnetic resonance** Undefined	**Cardiac magnetic resonance** ^a^ Change in ECV (≥5% decrease)
	**Nuclear imaging** Bone scintigraphy represents the pillar of the non-biopsy diagnostic algorithm for patients without MP. *(Three tracers labelled with 99mTc: pyrophosphate [PYP], 3,3-diphosphono-1,2 propanodicarboxylic acid [DPD] and hydroxymethylene diphosphonate [HMDP])*	**Nuclear imaging** Undefined	

AL-CA, light chain amyloidosis with cardiac involvement; ATTR-CA, transthyretin amyloidosis with cardiac involvement; ATTRwt, wild-type transthyretin amyloidosis; ATTRv, hereditary transthyretin amyloidosis; GLS, global longitudinal strain; T1, native T1; ECV, extracellular volume; PET, positron emission tomography; CT, computed tomography; NT-proBNP, N-terminal pro-B-type natriuretic peptide; hs-TnT, high sensitivity troponin T; NAC, National Amyloidosis Center; LVEF, left ventricular ejection fraction.

^a^Not yet universally accepted/proposed.

The multifaceted landscape of systemic amyloidoses is still characterized by a net distinction in terms of diagnostic approaches between cardiologists and internal medicine specialists or haematologists. Different backgrounds and expertise (cardiologists more confident with ATTR amyloidosis, haematologists with AL amyloidosis) may in part justify this. However, it should be recognized that an imaging-centred approach has often ended up dominating cardiologists’ actions both in the diagnostic workup and subsequent clinical management of the disease. This cultural behaviour is also reflected by the diagnostic algorithm of the ESC which suggests, in patients with suspected CA, the simultaneous performing of bone scintigraphy and haematologic tests, while the imaging test must follow the exclusion of a MP whose presence always requires (at present time) the histological confirmation/exclusion of AL amyloidosis.^17,1^ Current approach might potentially favour diagnostic delays and errors, with ascertained negative prognostic implications for patients.^3^ If on the one hand, a non-biopsy imaging-based diagnostic algorithm applicable to all the systemic amyloidoses and able to differentiate phenocopies in patients presenting with ‘hypertrophic cardiomyopathy’ represents a primary goal of research (in this context radionuclide imaging might represent a game changer, although the available experiences need to be confirmed in larger studies with external validation cohorts), we should acknowledge current limitations.^58^ These regard not only diagnosis but also the assessment of disease progression and response to treatments. Cardiac biomarkers might, at least in part, overcome these limitations, since they may be more sensitive in the subclinical phases of the disease (albeit at the cost of decreased specificity being influenced by several factors including fluid balance and status, atrial fibrillation [AF], and comorbidities), more accessible, less expensive, and usually more reproducible.

### Current limitations and future perspectives

**Figure 2:**
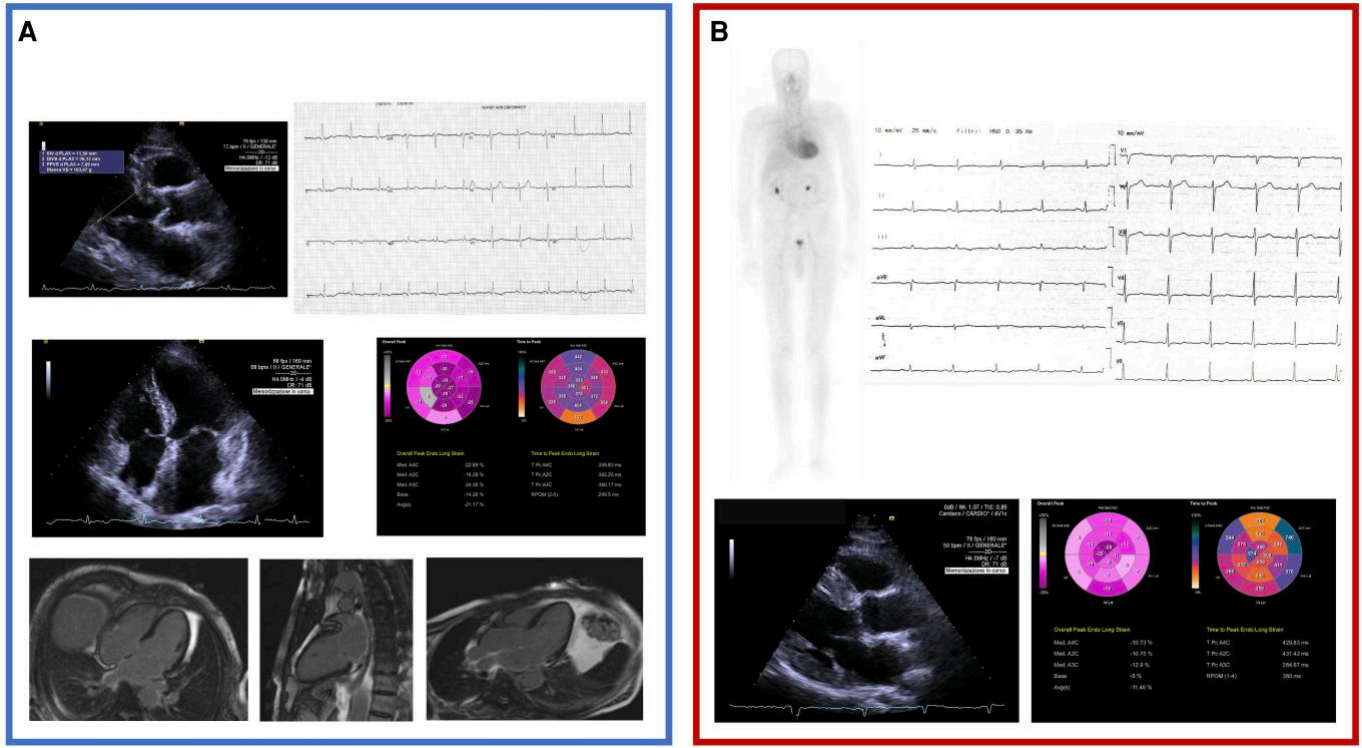
Strengths and limitations of cardiac biomarkers in the detection of preclinical AL-CA and ATTR-CA. (*A*) A 70-year-old man with multiple myeloma recently diagnosed with AL-CA following an increase in hs-cTnI and NT-proBNP. His past medical history included arterial hypertension with optimal blood pressure control (without orthostatic hypotension). The echocardiogram showed borderline-increased wall thickness (interventricular septum thickness—IVSd 11–12 mm) with preserved left ventricular ejection fraction, no significant diastolic dysfunction, and left atrial dilatation. Cardiac magnetic resonance imaging did not show late-gadolinium enhancement. Cardiac biomarkers were increased: hs-cTnI 78 ng/L (upper limit 57 ng/L), NT-proBNP 1968 ng/L. (*B*) An 87-year-old man with ATTRwt-CA. His diagnosis was incidental after an echocardiogram (ECG) was performed for cardiovascular prevention. ECG showed borderline-reduced QRS voltages in the peripheral leads with abnormal voltage-to-mass ratio. It also showed markedly increased wall thickness (IVSd 20 mm) with preserved left ventricular ejection fraction. ^99m^Tc-DPD bone scintigraphy revealed intense cardiac uptake (Perugini score 2). Cardiac biomarkers were within normal reference ranges: hs-cTnI 21 ng/L (upper limit 57 ng/L), NT-proBNP 189 ng/L. The patient had no HF symptoms, and he was not treated with diuretics.

Despite several advantages, cardiac biomarkers have limitations. First, although they are more sensitive in the early detection of AL amyloidosis or possibly in TTR mutation carriers, this may be not necessarily true in patients with ATTRwt-CA. With the increased awareness of the disease, it is possible to intercept with non-invasive imaging tests patients in the preclinical phase, despite the presence of cardiac involvement (e.g. patients with prostate cancer undergoing bone scintigraphy to exclude metastases, those undergoing echocardiographic evaluation due to the presence of red flags such as carpal tunnel syndrome or lumbar spinal canal stenosis).^20^ These patients may exhibit phenotypic cardiac abnormalities (e.g. increased wall thickness, abnormal strain pattern) with both NPs and troponins within normal limits (*Figure 2*). Another significant limitation is represented by the lack of standardized cutoffs in patients with suspected ATTR-CA.^14^ The concentration of NPs can be influenced by comorbidities and physiological variables that can increase (e.g. age, AF, reduced eGFR, diabetic ketosis) or decrease (obesity) their level thus interfering with clinical interpretation. Some of these variables are not uncommon in patients with CA.^59^ In patients with advanced chronic kidney disease—CKD (i.e. stages 4–5), NPs are markedly elevated and no established diagnostic thresholds exist. In this context, BNP might be a more accurate marker of cardiovascular risk due to NT-proBNP’s renal clearance.^60^ Although providing the same information BNP and NT-proBNP have specific cutoffs and they are not interchangeable; moreover, NT-proBNP is most commonly used for diagnostic purposes, cardiac staging, disease progression, and in clinical trials.^13,22^ This might represent a potential obstacle whenever only BNP is available, although specific staging systems and cardiac response criteria have been developed using this NP.^61,62^ Regarding troponins, it should be kept in mind that differences exist between hs-cTnI and hs-cTnT, and that is crucial to consider the vendor-specific assay platform when comparing values in clinical practice and research studies.^14^ There are key differences in reported 99th reference percentile, limit of detection, and the lowest concentration at which a 10% coefficient of variation is obtained between platforms.^14^ Finally, cardiac biomarkers are influenced (as mentioned above) by coexistent cardiac conditions other than amyloidosis, and this still causes uncertainty when interpreting the laboratory findings. Despite the aforementioned limitations, in the next future, we hope to find universally acceptable thresholds of cardiac biomarkers to confidently rule out ATTR-CA. In patients with ATTR-CA, changes in serum TTR levels and the use of TTR aggregate detector (TAD1) might become standards to monitor disease progression and treatment response together classical laboratory biomarkers.^41,56^

## Learning points

1. Cardiac biomarkers represent an invaluable tool in the diagnosis and management of patients with CA.
2. Cardiac biomarkers (namely NPs and troponins) can be more sensitive than imaging techniques in the early detection of AL-CA.
3. Cardiac biomarkers still represent the best way to monitor organ response in AL-CA, and one of the main parameters to evaluate treatment response.
4. Changes in serum TTR levels seem to be promising to monitor treatment response in ATTR-CA.
5. Emerging biomarkers (e.g. TAD1 for ATTR amyloidosis, hepatocyte growth factor [HGF], growth differentiation factor-15 [GDF-15], endothelin—1 [ET-1], etc) should be used for early detection, differential diagnosis, and prognostic purposes but also for monitoring treatment response.
6. At present time, cardiac imaging integrates the clinical information provided by cardiac biomarkers, but it cannot replace laboratory information.
7. Further larger studies will help confirm the ability of cardiac imaging (e.g. GLS with echo, ECV, and native T1 with CMR) to refine current staging systems and to track changes useful to define treatment response.
8. Preliminary data suggest a potential role of radionuclide imaging in correctly differentiating not AL-CA from ATTR-CA and other forms of ‘cardiac hypertrophy’, but larger confirmatory studies are needed. This suggests caution before validating some tracers in a non-invasive diagnostic workup.

To conclude, cardiac biomarkers are able to unveil the unseen, helping physicians to navigate beyond the horizons of imaging. The core message remains the need for integration of biomarkers and imaging as this kind of approach gave us the best results with a clear indication of the route to follow. To quote Vincent van Gogh, ‘*now that the leaves have fallen, the countryside resembles that of the North, and I realize that if I went back there, I would see things much clearer than before*’.

## Lead author biography

Dr. Giuseppe D. Sanna serves as a consultant cardiologist at Sassari University Hospital in Italy. His specialist training included a fellowship at the Echocardiology lab of the Hammersmith Hospital (London, UK) (2012–2013) and a fellowship at the CMR Unit of the Heart Hospital (London, UK) (2014). In 2021, he completed the Postgraduate Course in Heart Failure (PCHF) (London, UK). He received a Cardiac ATTR Amyloidosis Fellowship grant funded by the International Society of Amyloidosis and worked as a researcher at the Amyloidosis Research and Treatment Center (University of Pavia) in 2024. His clinical and research activities include HF and cardiomyopathies, cardiovascular imaging, and cardiac pacing.
